# Blood extracellular vesicles from healthy individuals regulate hematopoietic stem cells as humans age

**DOI:** 10.1111/acel.13245

**Published:** 2020-10-07

**Authors:** Isabelle Grenier‐Pleau, Kathrin Tyryshkin, Tri Dung Le, John Rudan, Eric Bonneil, Pierre Thibault, Karen Zeng, Cecilia Lässer, David Mallinson, Dimitrios Lamprou, Jialui Hui, Lynne‐Marie Postovit, Edmond Y. W. Chan, Sheela A. Abraham

**Affiliations:** ^1^ Department of Biomedical and Molecular Sciences Queen's University Kingston ON Canada; ^2^ Department of Pathology and Molecular Medicine Queen's University Kingston ON Canada; ^3^ Department of Surgery Kingston Health Sciences Centre Queen's University Kingston ON Canada; ^4^ Proteomics and Bioanalytical Mass Spectrometry Research Unit Institute for Research in Immunology and Cancer of the Université de Montréal Montréal QC Canada; ^5^ Krefting Research Centre Institute of Medicine at Sahlgrenska Academy University of Gothenburg Gothenburg Sweden; ^6^ Strathclyde Institute of Pharmacy and Biomedical Sciences University of Strathclyde Glasgow UK; ^7^ School of Pharmacy Queen's University Belfast UK; ^8^ Department of Oncology University of Alberta Edmonton AB Canada

**Keywords:** aging, clonal hematopoiesis, exosomes, extracellular vesicles, hematopoiesis, hematopoietic stem cells

## Abstract

Hematopoietic stem cells (HSCs) maintain balanced blood cell production in a process called hematopoiesis. As humans age, their HSCs acquire mutations that allow some HSCs to disproportionately contribute to normal blood production. This process, known as age‐related clonal hematopoiesis, predisposes certain individuals to cancer, cardiovascular and pulmonary pathologies. There is a growing body of evidence suggesting that factors outside cells, such as extracellular vesicles (EVs), contribute to the disruption of stem cell homeostasis during aging. We have characterized blood EVs from humans and determined that they are remarkably consistent with respect to size, concentration, and total protein content, across healthy subjects aged 20–85 years. When analyzing EV protein composition from mass spectroscopy data, our machine‐learning‐based algorithms are able to distinguish EV proteins based on age and suggest that different cell types dominantly produce EVs released into the blood, which change over time. Importantly, our data show blood EVs from middle and older age groups (>40 years) significantly stimulate HSCs in contrast to untreated and EVs sourced from young subjects. Our study establishes for the first time that although EV particle size, concentration, and total protein content remain relatively consistent over an adult lifespan in humans, EV content evolves during aging and potentially influences HSC regulation.

## Graphical abstract

Despite hematopoietic stem cells losing functionality over time, the production of blood cells is continuously maintained as humans age. We report here that circulating extracellular vesicles are continuously produced over adulthood, but change in composition. Functional data support that individuals over 40 years possess extracellular vesicles that stimulate hematopoietic stem cell proliferation and potentially help support blood production as humans age.

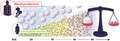

Hematopoietic stem cells (HSCs) are defined by their ability to self‐renew and differentiate into all blood and immune cell lineages (Weissman & Shizuru, 2008). HSCs are reliant on both cell autonomous and nonautonomous cues to balance self‐renewal and blood production within the bone marrow. Aging has profound effects on HSCs (Pang et al., 2017), leading to loss of regenerative capacity (Seita & Weissman, 2010), deficient DNA repair (Martin‐Pardillos et al., 2017), reduced telomere length (Halaschek‐Wiener et al., 2008), mitochondrial and metabolic deregulation (Mohrin & Chen, 2016) and the accumulation of somatic mutations leading to clonal hematopoiesis (CH) (Shlush, 2018; Steensma, 2018). It is critical to understand how HSC aging contributes to the development of hematologic disorders, especially in light of the elderly population exponentially expanding worldwide (WHO, 2017).

Hematopoietic stem cell regulation involves multiple factors provided by the bone marrow niche, that is in continuous free‐flow with the circulatory system (Lucas, 2017). Despite decreased functionality associated with aging, HSCs must maintain a production rate of 10^10^–10^11^ blood cells per day. This extraordinary demand highlights the importance intercellular communication mechanisms play, in coordinating HSC activity.

There is a lack of understanding regarding circulating extracellular signaling factors that contribute to HSC regulation, and how they evolve in the context of aging (Verovskaya et al., 2019). Extracellular vesicles (EVs), cell‐derived lipid membrane structures involved in intercellular communication, have been purported as key communication mediators between cells; however, there is a paucity of knowledge regarding how EVs contribute to HSC maintenance over time (Butler et al., 2018).

To determine if blood circulating EVs differ in humans of different ages, we characterized plasma‐sourced EVs from healthy young (20–39 years), middle‐aged (40–59 years), and older (60–85 years) individuals (*n* = 35). We enriched for vesicles, which we enumerated and immunophenotyped. Tetraspanin expression confirm commonly associated EV markers CD9, CD63 and CD81 are heterogeneously expressed among age groups (Figure S1), which was expected, based on our techniques, biological variability, and previous work confirming the highly irregular expression of these EV markers (Kowal et al., 2016; Thery et al., 2018).

Using nanoparticle tracking analyses with protein quantification, it was determined that averaged EV particle size (129.6 ± 2.1 nm), particle concentration (1.4 × 10^9^ ± 1.7 × 10^8^ particles/ml plasma), and protein concentration (1.9 × 10^−8^ ± 1.3 × 10^−9^ µg protein/per particle) remain consistent among all individuals (Figure 1a‐c, Figure S2A‐C). When data are re‐analyzed by sex, no quantifiable differences were observed in all EV parameters measured (Figure 1d‐f; Figure S3A‐C). Results suggest that blood EVs, similar to other blood parameters (e.g., red and white blood cells, electrolytes, oxygen concentration) have a physiological range that can be reproducibly quantitated in healthy subjects, and do not significantly change during the course of adult life. Results presented here differ from earlier reports (Eitan et al., 2017) suggesting EVs decrease with age; however, we believe observed differences presented here are based on our 2‐part EV enrichment, that mostly excludes contaminating particles such as lipoproteins and chylomicrons (Karimi et al., 2018) that may vary with age.

**Figure 1:**
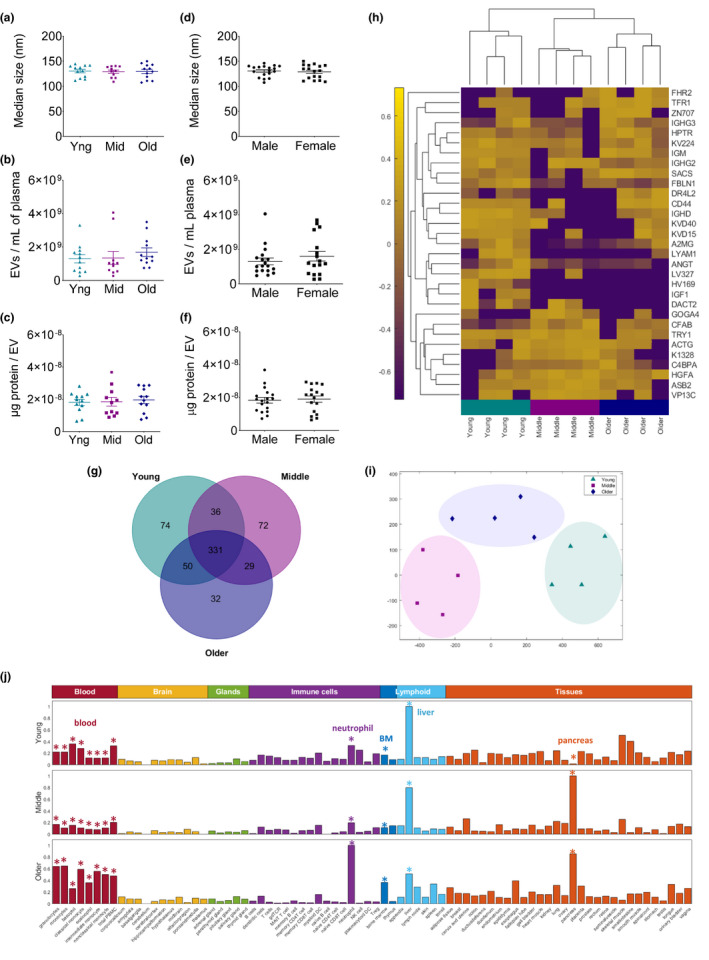
Characterization of blood EVs from healthy subjects aged 20–85 years. (a) EV particle size, (b) EV concentration per ml plasma and (c) protein per particle from young (20–39 years), middle (40–59 years), and older (60–85 years) subjects (*n* = 35). Subjects grouped by age or (d‐f) sex. Particle size/concentration determined by nanoparticle tracking analysis (ZetaView^®^), protein determined using Qubit^®^. Statistical analyses between age groups (young: *n* = 12, middle: *n* = 11, older: *n* = 12): ordinary One‐Way ANOVA, sex groups (male: *n* = 18, female: *n* = 17): an unpaired *t*‐test. (g) Venn diagram of median EV protein expression analyzed by LC‐MS/MS sourced from young, middle, and older individuals. (h) Heatmap of age discriminating proteins using unsupervised selection algorithm analyzed using hierarchical clustering. (i) t‐Distributed stochastic neighbor embedding (t‐SNE) plot using age discriminating proteins. (j) Top 5% EV proteins from each age group cross‐referenced to the Human Protein Atlas (https://www.proteinatlas.org/humanproteome/tissue)

To further characterize blood EVs, we performed unbiased quantitative proteomic studies on trypsin‐digested EV proteins from subjects representing the three age groups (*n* = 12 total). The median protein expression of each age group defined age‐specific and overlapping profiles (Figure 1g; Table S2). Machine‐learning techniques were used to identify and rank proteins on their ability to discriminate between age groups, with an established feature selection algorithm (Ren et al., 2017) with leave‐one‐out validation. This algorithm ranks proteins based on their ability to discriminate between two groups, which we have shown, efficiently identifies molecular patterns (Panarelli et al., 2019, Turashvili et al., 2018). Unsupervised hierarchical clustering analyses using the top ranking 5% proteins revealed that young, middle, and older subject EV proteins independently group, having distinguishably defining protein signatures (Figure 1h,i) that are independent of sex (Table S3).

Because EV content has been confirmed to reflect parental or the donor cell source (Kucharzewska et al., 2013; Sancho‐Albero et al., 2019) we cross‐referenced our EV proteomic dataset using the top 5% of ranked proteins in each age group, with the human protein atlas (Uhlen et al., 2015) to identify potential cell sources of circulating EVs. Our in silico results suggest that as humans age, circulating EVs are dominantly produced by bone marrow and blood cells that include monocytes and granulocytes, namely neutrophils. EVs originating from the liver decrease over time, with increasing levels of EVs released from the pancreas as humans age (Figure 1j).

To ascertain if circulating blood EVs impart functional effects on HSCs with respect to proliferation and differentiation capacity, in vitro colony‐forming cell (CFC) assays were performed using cord blood sourced CD34^+^ (hematopoietic stem and progenitor cells (HSPC). HSPC cells were incubated with EVs (at a concentration 1 log‐fold lower than our estimated circulating EV concentration in plasma (Figure S2B) from the different age groups, for 48 h followed by plating cells in methylcellulose media with defined growth factors. Results indicate EVs from middle and older‐aged subjects increase the colony‐forming capacity of CD34^+^ HSCs relative to EVs from younger individuals (Figure 2a‐c). Replicate experiments using differing EV sources (*n* = 12) and cord blood samples (*n* = 9) support that EVs from all subjects over 40 years of age significantly stimulate HSCs. This stimulatory effect is not limited to cord blood HSCs, but can be observed using CD34^+^ cells enriched from older individuals (42–80 years). When quantifying colony types, no statistically significant differences were observed in the types of colonies produced, suggesting that increased colonies was a result of stimulating primitive progenitors (i.e., CFU‐GEMM Figure 2a) rather than the EVs augmenting differentiation. Staining HSPC with CD34 and CD38 fluorescently conjugated antibodies, after culturing the cells with EVs (under the same conditions as Figure 2a‐f) for 48 h, provide evidence that EVs from older individuals stimulate expansion of the multipotent progenitor population, as assessed by flow cytometry, which is growth factor independent (Figure 2g). These data reveal that middle and older subjects have circulating EVs capable of stimulating primitive HSCs.

**Figure 2:**
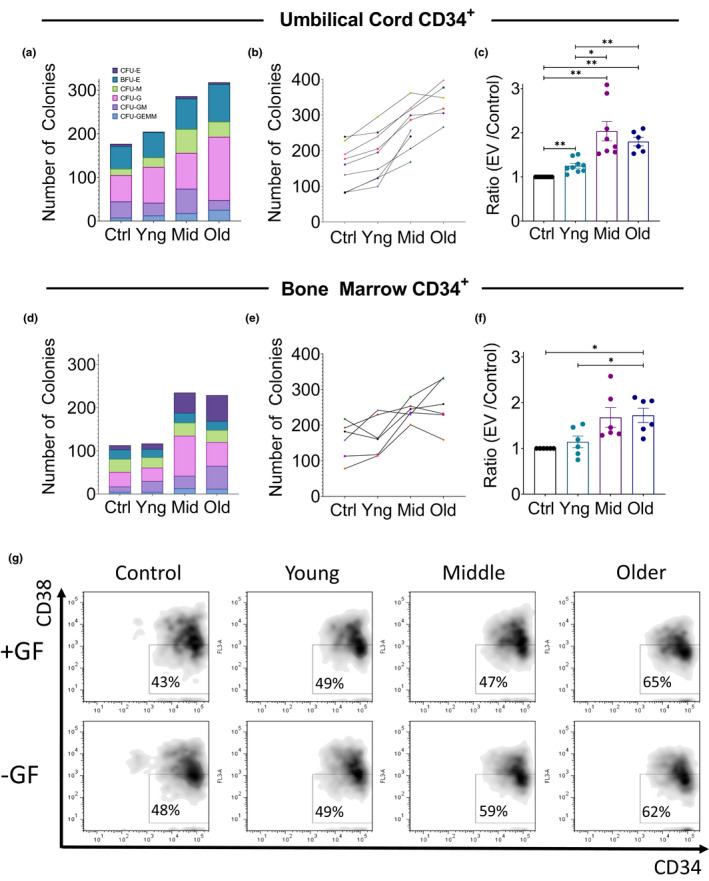
Blood EVs from middle‐aged and older subjects stimulate hematopoietic stem cells. Colony‐forming Cell (CFC) assays using cord blood CD34^+^ cells cultured in serum‐free media (+IL‐3, IL‐6, G‐CSF, Flt‐3, stem cell factor) and incubated with EVs or not (PBS control) for 48 h. Data presented as (a) representative experiment (b) all experiments young (*n* = 9), middle (*n* = 8), older (*n* = 6) and (c) normalized averaged experiments. (d‐f) CFC assays as above using bone marrow CD34+ cells (42–80 year) with EVs sourced from indicated age young (*n* = 6), middle (*n* = 6), old (*n* = 6). Statistical analysis: mixed‐effects analysis, with Geisser–Greenhouse correction, along with Tukey's multiple comparisons test with individual variances computed for each comparison. *p < 0.05, **p < 0.01. (g) Flow cytometry of the HSPC treated as above, with or without growth factors. Samples were stained with CD34 and CD38 antibody, gates defined by isotype controls

The present study demonstrates that healthy individuals maintain circulating EVs consistent in terms of size, particle concentration, and total protein per particle between 20‐85 years of age. In contrast, blood EV protein profile composition changes over time in humans, and our in silico analyses, suggests that certain organs/ cell types may be responsible for this change, by altering EV production and releasing EVs into the bloodstream. Most strikingly, the blood circulating EVs produced from middle and older‐aged individuals stimulate HSC colony‐forming ability in contrast to younger individuals and untreated controls. This work highlights that blood EVs impart important extracellular signals to HSCs as humans age. We posit that EVs may provide a compensatory stimulus that counter‐balances a decrease in HSC functionality in individuals approaching middle‐age. Current work is investigating how these activation signals, provided by the EVs, may impact the clonal trajectory of mutated CH clones, which may have profound implications in the development of hematological‐based malignancies later in life. We demonstrate, for the first time a fundamental age‐specific difference in blood EVs that specifically affects HSCs in individuals after 40 years of age, prior to the detection of classically defined clonal hematopoiesis.

## CONFLICT OF INTEREST

The authors declare that there are no competing financial interests.

## AUTHOR CONTRIBUTIONS

IGP recruited blood donors, performed experiments, and analyzed data and developed figures. KT analyzed data and developed figures. TDL assisted with biological experiments. JR isolated and provided primary bone marrow samples. EB and PT performed mass spectrometry analyses and support. KZ and EYWC provided western blot analyses. JH and LMP provided electron microscopy analyses. DM and DL contributed to EV characterization with validation of size and morphology using microscopy. CL provided support with developing EV enrichment methodology. SA designed the study, performed experiments, and analyzed data. All authors contributed to manuscript preparation and revision.

## Data Availability

Data available upon request and will be deposited in figshare.
