# Supplementary material for: Blood extracellular vesicles from healthy individuals regulate hematopoietic stem cells as humans age
Source: Aging Cell. 2020 Oct 7;19(11):e13245. doi: 10.1111/acel.13245 (PMC7681054; doi:10.1111/acel.13245)
Supplement: Supplementary file 1 [file ACEL-19-e13245-s001.pdf]

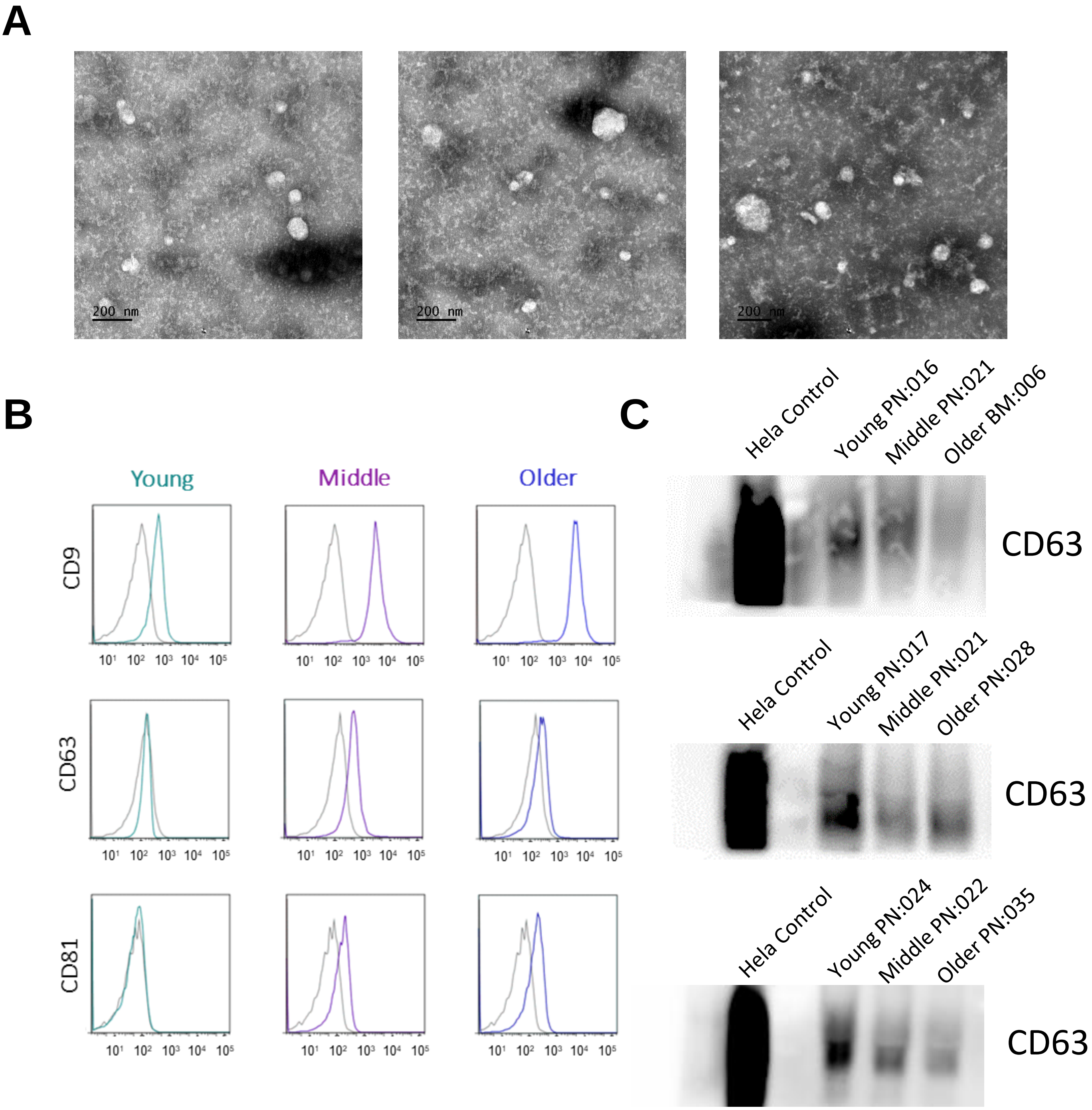

Supplemental Figure. 1 Physical characterization of extracellular vesicle size, shape, and tetraspanin expression among healthy subjects. (**A**) Representative electron microscopy images of EVs (**B**) Tetraspanin expression of blood EVs varies among different aged healthy subjects. Tetraspanin positive populations identified using anti-CD9, anti-CD63, or anti-CD81-coated superparamagnetic polystyrene beads (4.5μm) that were incubated with EVs from young (20-39 years), middle (40-59 years), or older (60-85 years) subjects. EV-captured beads were immunostained with CD9 antibody and compared with isotype control (grey). **(C)** Western blot of tetraspanin CD63 in young, middle-aged, and older subjects.

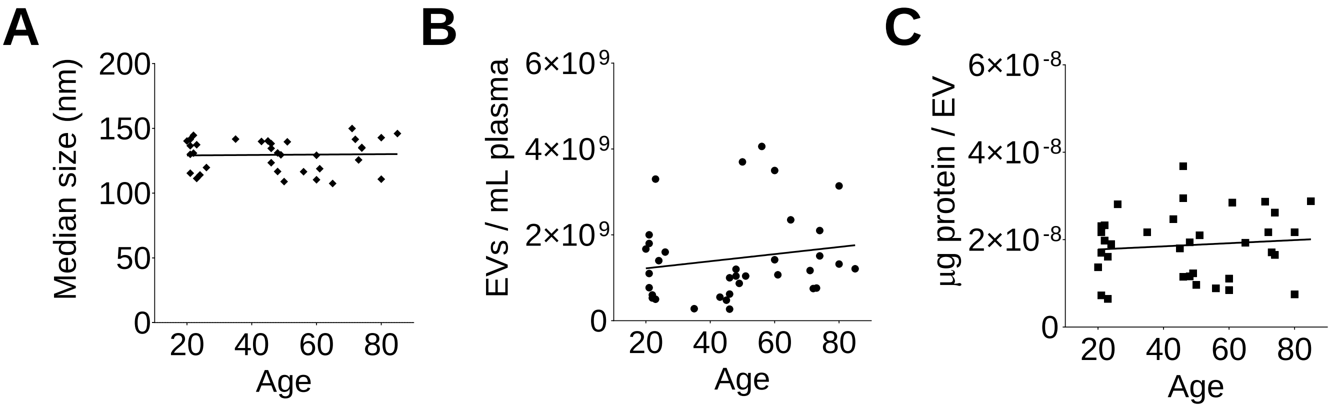

Supplemental Figure 2. Quantitation of blood extracellular vesicles (EVs) from healthy subjects aged 20-85 years. (A) Extracellular vesicle median particle size (**B**) EV concentration per ml plasma (**C**) Protein concentration per particle. Statistical analysis using linear regression analysis (n=35).

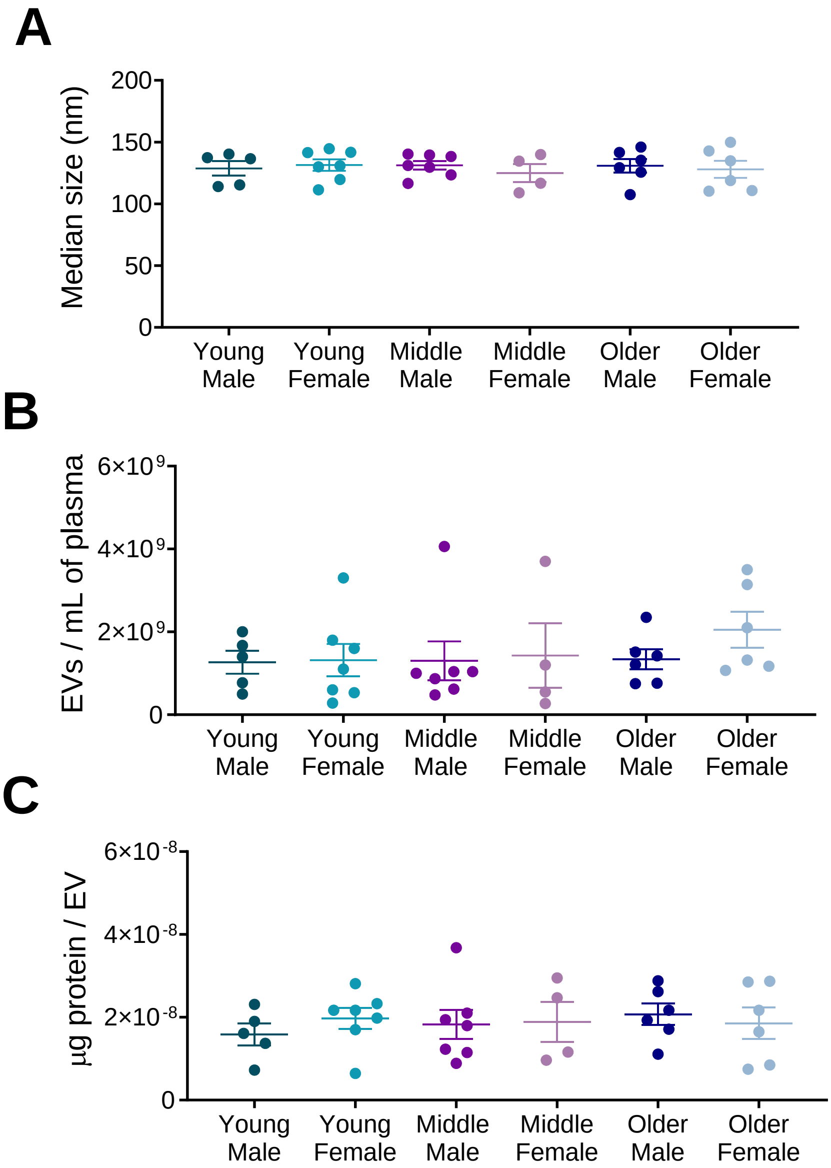

Supplemental Figure 3. Sex and age do not alter blood extracellular vesicle size, concentration or total protein per particle from healthy subjects (A) Extracellular vesicle particle size, (B) EV concentration per mL plasma and (C) protein per particle was determined from young (20-39 years), middle (40-59 years), and older (60-85 years) subjects (n=35).

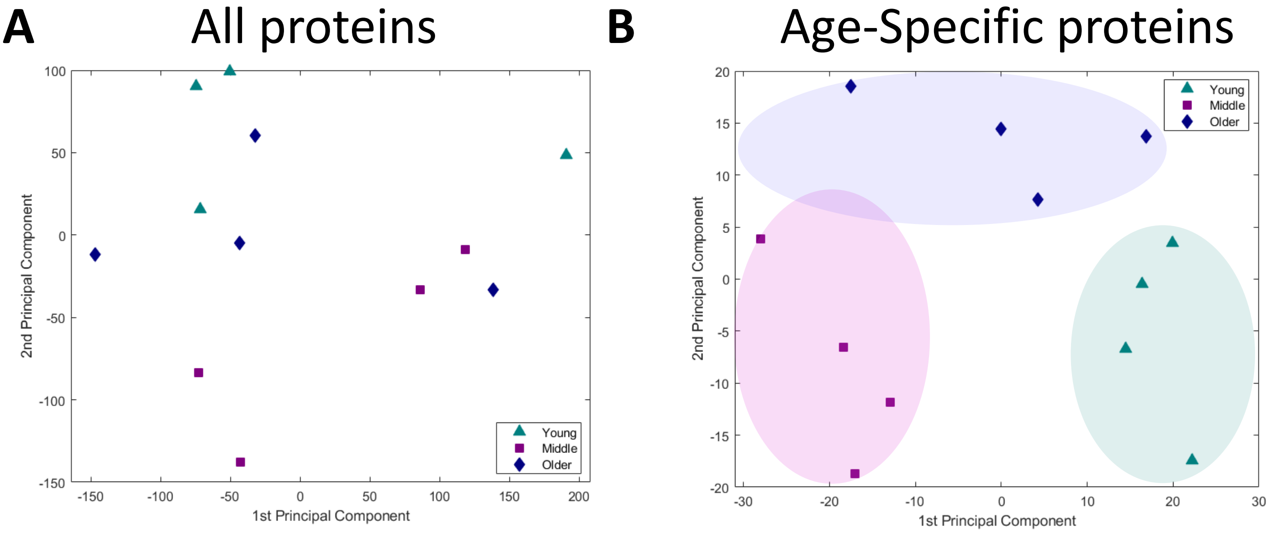

Supplemental Figure 4. Principal Component Analysis (PCA) of top ranked proteins from each age group. The 31 proteins chosen by a selection algorithm using machine learning techniques that best discriminate young, middle, and older EVs from one another were used for PCA (n=12 biological samples).

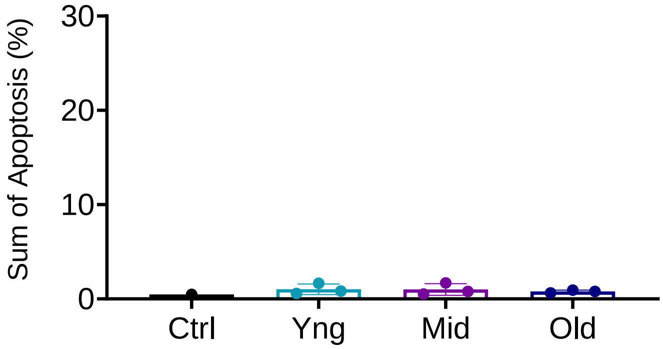

Supplemental Figure 5. Apoptosis is undetectable in CD34$^+$ hematopoietic stem cells after treatment with EVs from various age groups. Apoptosis levels were quantitated (as described in the methods) in HSPCs after incubation with EVs post 48 hours (n=3).

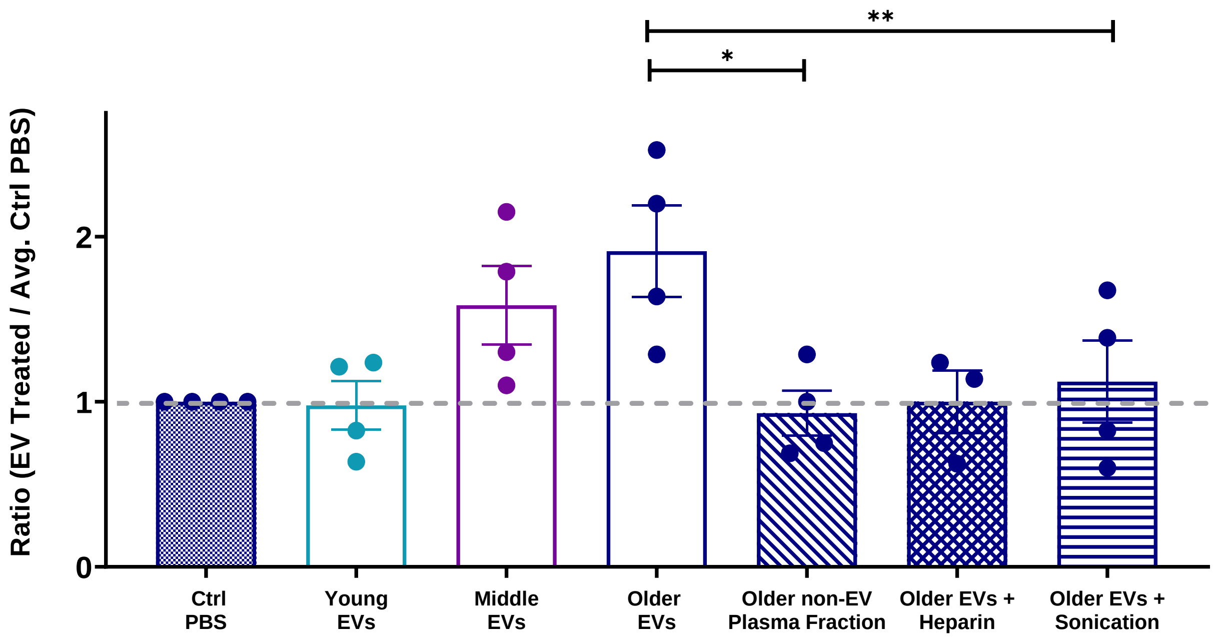

Supplemental Figure 6. Non EV containing plasma fractions, heparin treated EVs or sonicated EVs do not stimulate hematopoietic stem cells. CFC assays (as described in the methods) using umbilical cord samples. Columns represent no EVs (Ctrl PBS), young, middle or older (n=4 per treatment) sourced EVs, non-EV plasma fraction from older subjects (n=4), older EVs incubated with heparin (100µg/mL) (n=3) or older EVs sonicated (n=4). Statistical analysis: mixed-effects analysis, with Geisser-Greenhouse correction, along with Tukey's multiple comparisons test with individual variances computed for each comparison (*$p < 0.05$, **$p < 0.01$).

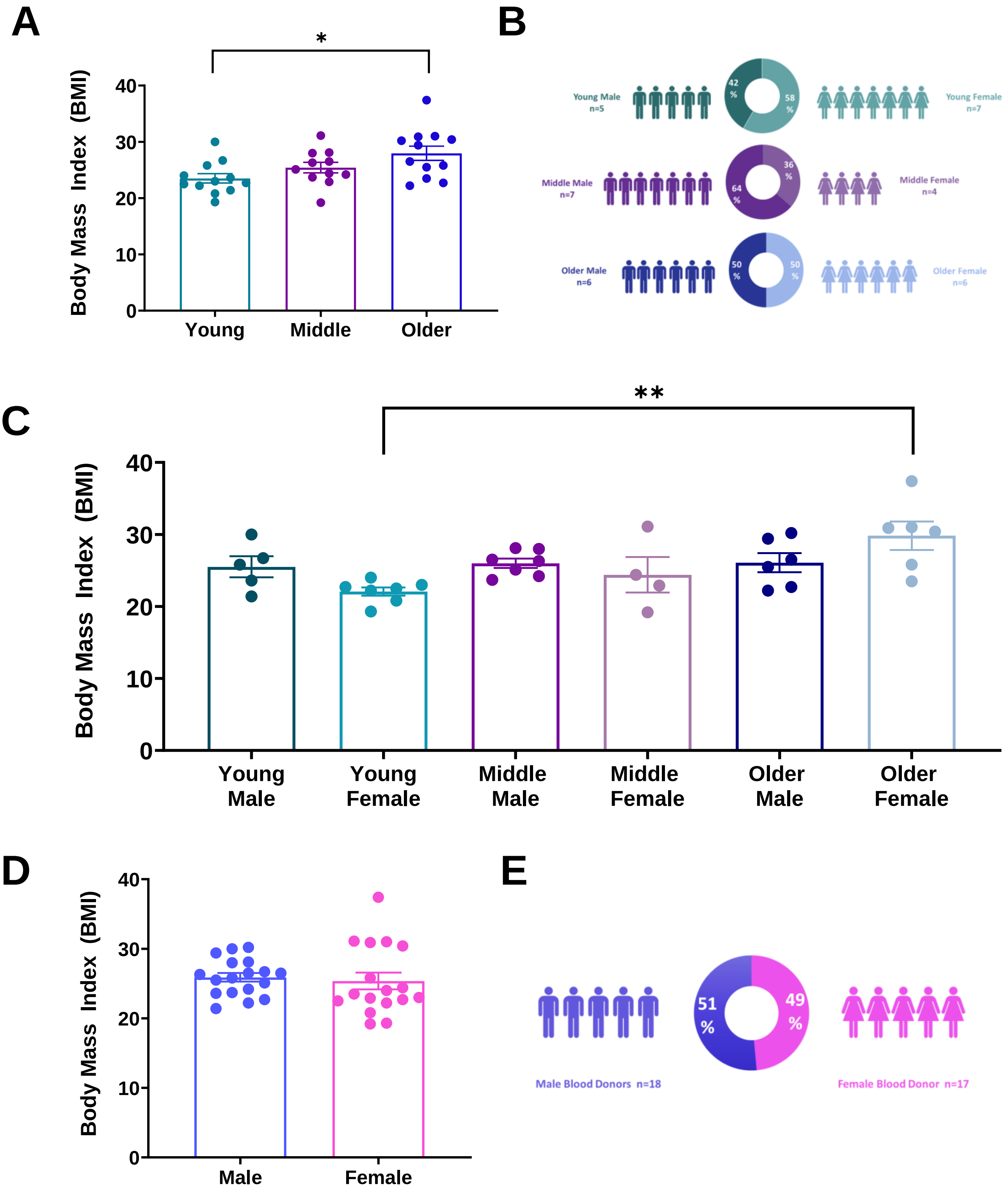

Supplemental Figure 7. Body Mass Index (BMI) of study subjects. (A) BMI of 35 adult blood donors separated into young, middle, and older age groups **(B)** and sex. **(C)** BMI of age groups, additionally separated by sex **(D)** BMI comparison of total adult male and female subjects. **(E)** Distribution of male and female participants in study (males=18 females=17). Statistical analysis: Kruskal-Wallis test, along with Dunn's multiple compassion test (*p < 0.05, **p < 0.01).

Supplemental Table 1. Medical History of blood donors for EV enrichment (n=35)

	Patient Number	Sex	Age	BMI	Ethnicity	Disease/Health conditions	Anticoagulant treatment	Smoking History
Young (20-39 yr)	PN 002	Female	22	22.2	Métis	None	No	No
	PN012	Male	23	25.8	Caucasian	None	No	No
	PN 013	Male	24	21.4	Caucasian	None	No	No
	PN 015	Female	22	22.5	Caucasian	None	No	No
	PN 016	Female	23	24	Caucasian	None	No	No
	PN 017	Female	21	19.3	Caucasian	None	No	No
	PN 018	Female	35	20.8	Asian/Caucasian	None	No	No
	PN 019	Male	21	30	Caucasian	None	No	No
	PN 023	Female	26	23	Caucasian	Leukopenia and anemia	No	No
	PN 024	Female	21	22.7	Caucasian	None	No	No
	PN 025	Male	20	23.6	Asian	None	No	No
	PN 036	Male	21	26.7	Caucasian	None	No	No
Middle-aged (40-59 yr)	PN 001	Female	48	22.9	Asian (Indian)	None	No	No
	PN 005	Male	46	24.2	Asian	None	No	No
	PN 006	Female	46	19.2	Caucasian	iron deficiency	No	No
	PN 007	Male	46	26.5	Caucasian	None	No	No
	PN 010	Male	45	28.1	Caucasian	None	No	Yes
	PN 020	Male	49	25.1	Caucasian	None	No	No
	PN 021	Male	48	28	Caucasian	None	No	No
	PN 022	Female	43	31.1	Caucasian	None	No	No
	PN 026	Female	50	24.4	Asian	None	No	No
	PN 027	Male	51	23.7	Asian (east asian)	Severe latex allergy	No	No
	PN 030	Male	56	26.3	Asian (Chinese)	Mild hypertension, high cholesterol	Yes (aspirin)	No
Older (60-85 yr)	PN 028	Male	65	25.5	Caucasian	None	No	No
	PN 029	Male	72	22.2	Caucasian	None	No	No
	PN 031	Male	60	22.7	Métis	None	No	No
	PN 032	Male	74	26.5	Caucasian	Tuberculosis (1952), Meningitis (1956), gallbladder polyps (2019)	No	Yes
	PN 033	Female	60	30.9	Caucasian	None	No	No
	PN 034	Female	61	30.4	Caucasian	Asthma, sleep apnea	No	No
	PN 035	Female	80	25.8	Caucasian	Thyroid gland	No	No
	BM 006	Female	71	23.5	Caucasian	None	No	No
	BM 007	Male	73	29.4	Caucasian	Metastatic melanoma (2017) in remission	No	No
	BM 012	Female	80	37.4	Caucasian	None	Yes (aspirin)	No
	BM 014	Male	85	30.2	Unknown	Anemia, thrombocytopenia, etc.	Unknown	Yes
	BM 016	Female	74	31	Caucasian	None	No	Yes

Supplemental Table 2. Venn diagram proteomic extracellular vesicle content by age

**Young, Middle & Older**	SPTN4,MA2B1,K1522,URP2,ADSV,KV116,A1BG,F13A,K1328,C1QA,TETN,IGHA1,MYH9,O10W1,VTDB,MAP4,CAN1,APOC2,A2AP,SHRM3,GTR1,IGL1,CPN2,IGKC,HRG,HGFL,CD5L,ITA2B,SH3L3,ATRN,PDIA3,C1RL,PRAF3,PGRP2,KVD28,ANGL6,RIC3,GP1BB,TTHY,MYL9,RAP1B,TBA8,1433Z,PPIF,PYRG2,TPM3,LV319,IC1,LOX12,RAB7A,TSP1,CAPZB,PRG4,SFRP5,APOH,PI16,TYB4,SYUA,SRCRL,ITB1,SAA4,K1C23,KVD20,ALS,CO6,RB8NL,IGF2,TFR1,FETUB,C1S,PLMN,ML12B,HEG1,PLF4,SEM6A,ANT3,ABLM3,PZP,IGHM,MTF2,HV601,PDLI1,FCN3,ITIH3,BIP,KVD21,ZYX,HV349,KNG1,IGLL1,DCTN2,HGFA,FBLN1,ZSWM9,VASP,ILK,CAP1,C4BPA,RB11B,ASB2,HNRPQ,APOC3,PROS,AFAM,VTNC,MCCA,MYH14,CLUS,THRB,SPTB1,PLPL8,HV313,APOF,FRIL,HEMO,TRIM7,HSP7C,HELZ2,HV366,HBB,PEDF,GPV,MMRN1,KV320,SAMP,TLN1,FCGBP,NOS1,TNG6,DYH5,ALBU,FA5,VINC,PPIA,TPM1,APOD,FETUA,TRY1,RET4,COF1,EPOP,HV364,C4BPB,LUM,LDHA,HV551,PIGR,HEP2,RAB1B,ECM1,TPIS,FHR1,K2C1,MOES,AACT,APOA,MASP1,CERU,ATPA,TENA,IGHG2,C1QC,APOL1,HPT,GP1BA,KVD11,APOC4,SYNE1,FIBG,GNAI2,COR1A,IGHD,SPRC,TRFE,CO3,ACTC,RAB10,RSU1,PRDX6,ACTN1,CO5,CATA,EHD3,CO4B,HSPB1,KLKB1,CAVN2,USH2A,TBA4A,FA12,DDR1,APOA2,C1QB,CBPN,IGM,C1R,GPIX,G3P,HV374,ENOA,ZA2G,PLEK,ARPC3,CO9,CXCL7,APOE,CALD1,COR1C,ABCCB,APOB,TAGL2,AT2A1,A2GL,HBD,HV307,FINC,CO8A,AKP13,HV372,CFAH,LG3BP,ACTB,KPYM,CO4A,ITIH2,HBA,BIG3,IGG1,PON1,LV147,VATC1,APOA1,SRGN,CO8G,MEF2B,FIBB,LVX54,DOPO,HABP2,NP1L1,HPTR,LV140,PROF1,S10A9,RASEF,APOC1,CC056,CO8B,KVD33,AMBP,DIAP1,GBB2,1433T,FLNA,CBG,WWOX,1433S,1433E,PRDX2,IGA2,VP13C,S10A4,COL11,PROP,TBB5,IGHG4,EPHA8,ATPB,CFAI,ITIH1,COX41,LV310,MYL6,CTF1,LV545,LTBP1,ANGT,A2MG,TERA,ACTG,ZSWM5,DEMA,CLH1,CSRP1,TRY3,GELS,CHLE,ALDOA,A1AT,STOM,KJ01,FA9,HV373,ITB3,K1C10,IGLC3,GBB1,STXB5,CO7,ARP3,K1C9,HV459,A1AG1,FCN2,CFAB,IGJ,TBB1,RP1,ATS13,FETA,TPM4,ITIH4,APOA4,KV401,DCD,APOM,PGK1,CO2,KV224,IGHG3,SYYM,PECA1,PGM1,HV315,TYB10,VWF,CLIC1,FIBA,B3AT,SACS,STX18
**Middle & Older**	TCPG,CNST,BIN2,ASAP1,MDHM,ANK1,RUS1,DEF1,CAZA2,ICAM2,ILEU,JPH3,KAIN,IGLC7,COR1B,PGAM2,TIMP1,CDC42,AT2A3,B2MG,LDHB,MARE1,RHOA,RASA3,RAC2,THSD1,1433G,SNP23,LASP1
**Young & Middle**	GLU2B,GANAB,MIF,SPAT7,CAZA1,YETS2,GRDN,41,UB2V1,G6B,DACT2,MBL2,PTN6,DAAM1,LRRF2,PARVB,SUGP2,WDR1,LV460,RAB21,CLCA,GOGA1,RTN4,ARPC4,DIDO1,CLCN3,ARP2,HP1B3,KAP0,GTR3,PARK7,WDR43,RAC1,ZN439,PPIP2,LV746
**Young & Old**	PHLD,ASXL2,KVD15,KV109,RTL1,KV105,LV144,RALB,KV139,FA10,KVD40,GDIR2,LV321,F13B,TRI10,LV861,HV226,COMP,LEG1,BAHC1,KI67,SAA1,T200C,CO1A1,EF1A1,S27A6,FNDC7,KV112,DR4L2,S39AC,RFXAP,A1AG2,TCF7,LV208,CD44,WDR35,LV151,OSBL1,ZN707,K22E,RHG09,SODM,CALX,LV39,HV5X1,LV469,VATA,CH60,AXA2L,K2C8
**Middle**	PSA1,RAB5C,SEPT7,ITA2,RB27B,ENPL,COTL1,NEUG,INT7,FCERG,STIM1,6PGD,TBB3,ZPI,TALDO,PRDX5,EPN4,SYYC,ARPC5,THBG,MDHC,ITA6,NID1,1433B,MARE2,IMB1,LPP,K1671,THTM,CASR,PFKAP,MYPT1,PI42A,K1C14,GOGA4,CAH2,EHD1,BTD,DP13B,RAB8A,CD36,BAP31,PRDX1,AT2A2,PDLI7,TYDP1,TBCA,TBA1A,FLOT1,1433F,PMS2,CYFP1,DREB,STX11,SRC8,CO6A1,TRFM,TPM2,BID,ROCK2,LRBA,GSTP1,RAB1A,DTD1,NEXN,PDIA6,TBB4A,SODC,CD59,FYB1,TBB2A,IBP3
**Older**	NEST,FCG3A,S10A6,TEC,RN111,PLEC,CTND1,2AAA,TRUB1,CD34,KALRN,FHR2,PDIA1,RANB9,NAA35,HV309,HS71A,ADT1,MCPH1,AMPN,DPP9,RBM25,PTPRQ,PCY1A,RHD,MRVI1,PGGHG,HV320,HV205,GXLT1,ICAM3,HBG2
**Young**	ANR17,PSA2,MACF1,ACSF4,RIMS1,SAC1,GPDM,HV146,KV106,KCNS1,IGF1,CALM2,TYPH,LV218,HV70D,KV230,SPART,LYAM1,HV311,MYO1F,DYH3,HV428,LV136,KV621,IGE,LV211,AMPD2,PNPH,AT1A1,KV127,MYO16,LIMS1,CPNS1,K2C6B,HV102,HS90A,GNA11,HV103,HV64D,NISCH,SORC1,FBX10,IGK,LV214,ABCA1,SPTA1,YLPM1,PRC1,ATIF1,COX5A,HV108,THAS,DOK3,KVD29,ATP5I,EFC13,EFL1,GGPPS,CH10,CPT1A,NMD3B,PRSR2,PIPSL,IVD,PACRL,SULF2,KV117,LV327,TF3C1,SEPP1,NPTX2,1A03,HV169,LYVE1

Supplemental Table 3. Age specific EV proteins are independent of sex

Protein Name	Kruskal Wallis test diff between sex (p-value)
IGHD	0.75
CD44	0.87
CFAB	0.52
HV169	0.70
LV327	0.59
FBLN1	0.34
KV224	0.26
KVD15	0.62
LYAM1	1.00
IGF1	0.53
ANGT	0.05
KVD40	0.51
HGFA	0.20
VP13C	0.87
TRY1	0.11
ACTG	0.75
A2MG	1.00
IGM	0.57
GOGA4	0.80
ASB2	0.34
K1328	0.87
C4BPA	0.63
SACS	0.42
ZN707	0.23
TFR1	0.42
HPTR	0.11
IGHG2	0.63
DACT2	0.44
DR4L2	0.93
IGHG3	0.75
FHR2	0.21

Supplemental Data: Detailed Methods

Ethics

All primary samples were collected from healthy individuals with no genetic conditions or active disease. Umbilical cord blood, bone marrow samples and blood samples were collected with the approval of the Queen's University Health Sciences and Affiliated Teaching Hospitals Research Ethics Board (HSREB) in Kingston, Canada (TRAQ# 602464). The HSREB operates in compliance with, and is constituted in accordance with, the requirement of the Tri-Council Policy (Canada). Statement: Ethical Conduct for Research Involving Humans (TCPS 2); the International Conference on Harmonisation Good Clinical Practice Consolidated Guideline (ICH GCP); Part C, Division 5 of the Food and Drug Regulations; Part 4 of the Natural Health Project Regulations; Part 3 of the Medical Devices Regulations, and the provisions of the Ontario Personal Health Information Protection Act (PHIPA 2004) and its applicable regulations.

Blood collection and plasma processing

The collection, handling and storage of blood extracellular vesicles followed guidelines outlined by the International Society for Extracellular Vesicles (ISEV) (Thery, C. (2018) J Extracell Vesicles. Nov 23;7(1):1535750). Peripheral blood was collected in the morning (9-11 am) from healthy subjects (20-85 yr) without fasting. For the isolation of plasma, blood was collected into K3E K3EDTA tubes (Greiner Bio-one), inverted and processed immediately. Blood was centrifuged at 1880×g / 10min / room temperature (RT). Plasma was transferred to 50mL polypropylene centrifuge tubes and centrifuged at 2500×g / 10 min / RT to remove platelets. Plasma was frozen at -80°C.

Isolation of extracellular vesicles by Iodixanol density cushion and size-exclusion chromatography

Frozen plasma was thawed at 37°C. To isolate EVs free of lipoproteins and chylomicrons we have used our published methodology (Karimi, N. et al. (2018) Cell. Mol. Life Sci. 75, 2873–2886). Briefly, a prepared iodixanol density gradient (OptiPrep Cedarlane, Canada) cushion was used in combination with size-exclusion chromatography (SEC). 6 mL volume of plasma was layered on top of a 2 mL 50% OptiPrep, 2 mL 30% OptiPrep, and 2 mL 10% OptiPrep cushion, in a 14x89mm polyallomer ultracentrifuge tube (Cedarlane, Canada), which was centrifuged at 178,000×g (SW 41 Ti rotor, k-factor 143.9, Beckman

Coulter, Brea, CA, USA) for 2 h at 4°C. A visible high-density band between the 10% and 30% layers was collected. Exactly 1ml of the high-density band was loaded onto a SEC column, which was prepared using Sepharose CL-2B (Sigma, USA) to a final volume of 10 mL, equilibrated with PBS. Sample was subsequently eluted with 10mL PBS and 0.5 mL fractions were collected, with fractions 7-11 (EV-enriched fractions) pooled together and stored in cryovials at -80°C.

Determination of size and concentration of extracellular vesicles

Particle median size and concentration of the pooled fractions were measured with a ZetaView® - Nanoparticle Tracking Video Microscope PMX-120 instrument according to the manufacturer's instruction (Particle Metrix, Germany). All samples were measured in triplicate, using identical instrument settings. The chamber temperature was measured and considered when size and concentration was calculated. Camera sensitivity was set to 80 and the shutter was set to 100. Data were analysed using the ZetaView Analyze software system, with a minimum particle size of 2.5nm, a maximum size of 6000nm, a minimum brightness of 30, a maximum brightness of 255, a minimum area of 10, and a maximum area of 1000. To validate particle size, atomic force microscopy (AFM) was completed with a selection of samples. Sample concentration was calculated (incorporating dilution factors) and indicated as particles per mL of plasma.

Extracellular vesicle protein quantification

To quantitate protein, the Qubit™ Protein Assay Kit (ThermoFisher Scientific, USA) was used, in conjunction with the Qubit® 3.0 Fluorometer (ThermoFisher, USA) as described in the manufacturer's instructions, standardized with PBS.

Preprocessing of umbilical cord blood and bone marrow

Umbilical Cord Blood (UBC) samples were obtained from healthy subjects minutes after caesarean section births at Kingston General Hospital, Canada. Umbilical cord was double clamped, and cord blood (4mL - 50mL) was drawn up in a 30mL syringe attached to an 18G needle containing citrate dextrose solution (12.5mL of anticoagulant for every 50mL of cord blood). Subsequently, cord blood was diluted (1:1) with PBS/0.1% Human Serum Albumin.

Bone Marrow (BM) samples (42-80 yr) were obtained from either total knee arthroplasty or total hip arthroplasty surgeries. Fresh BM was transferred to K3E K3EDTA tubes and inverted. Samples were subsequently diluted with PBS / 0.1% Human Serum Albumin and centrifuged at 300g / 10min / RT. Post

centrifugation, the upper fat layer was discarded, and the pellet was resuspended in 30mL of PBS / 0.1% Human Serum Albumin followed by two 300g / 7min /RT wash steps. After washes, the pellet was resuspended in 5-7 mL of PBS / 0.1% Human Serum Albumin depending on the original volume of bone marrow.

Isolation of $CD34^+$ cells from umbilical cord blood and bone marrow

$CD34^+$ cell enrichment followed within 20 minutes of sample collection. Either preprocessed UCB or BM samples were layered on top of the Ficoll-Paque Premium (Sigma, USA), and centrifuged at 1500rpm / 30min/ RT (Deceleration set to 0). Interface monocyte layer was collected, then diluted in 1:1 with PBS / 0.1% Human Serum Albumin and centrifuged 1500rpm / 10min / RT. Supernatant was discarded, and pellet was re-suspended in PBS / 0.1% Human Serum Albumin and centrifuged at 1200rpm / 10 min / RT twice. Positive selection of $CD34^+$ cells were completed using CD34 MicroBeads (Miltenyi Biotec), as per manufacture's instructions.

$CD34^+$ cell incubation with extracellular vesicles

Freshly enriched $CD34^+$ cells were cultured in serum free media consisting of Iscove's Modified Dulbecco's Medium (Sigma), a serum substitute B.I.T. (BSA/ Insulin/ Transferrin) (Stem Cell Technologies), M2-mercaptoethanol (Sigma), L-Glutamine, Penicillin/Streptomycin and Low density lipoprotein (Sigma). Media was supplemented (or not when indicated) with 100 ng/mL Flt3-ligand, 100 ng/mL stem cell factor, 20 ng/mL of Interleukin-3, 20 ng/mL of Interleukin- 6 and 20ng/mL granulocyte-colony stimulating factor (PeproTech). EVs from the indicated age groups were added to $CD34^+$ cells (final concentration of 2.3×10^4-2.5×10^5cells/mL) at a final EV concentration of 2.5-7.5 µg/mL in a round 96-well plate (total volume 200 µL) cultured for 48 hr at 37°C, 5% CO_2.

Heparin-binding incubation blocking EV uptake

EVs were incubated with 100 µg/ml of heparin (Sigma Aldrich, H3393 100-KU) overnight at 4°C, (Atai et al, 2013). Free heparin was removed using CL-2B mini spin columns.

Extracellular Vesicle Sonication

EVs were sonicated at high power for 20 second intervals on ice, with 1-minute rest, repeated 20 times and added to $CD34^+$ cells immediately.

Colony forming cell (CFC) assay

After culturing cells under various conditions as indicated, cells were washed with PBS / 0.1% Human Serum Albumin and centrifuged at 300g / 10min / RT. After counting viable cells using trypan blue dye exclusion, approximately 1000 $CD34^+$ cells were seeded into 1.2 mL of Methocult™ Medium H4435 Enriched (StemCell Technologies) and plated in 35mm plates using 16 gauge blunt-end needles followed by an incubation period of 10-14 days at 37°C and 5% CO_2. Colonies were subsequently both quantified and qualified according to their morphology as lineage-committed progenitor colonies (CFU-GEMM: Colony-Forming Unit- Granulocyte / Erythrocyte / Macrophage / Megakaryocyte; BFU-E: Burst-Forming Unit - Erythroid; CFU-E: Colony-Forming Unit – Erythroid; CFU-GM: Colony-Forming Unit - Granulocyte/Macrophage; CFU-G: Colony-Forming Unit – Granulocyte; CFU-M: Colony-Forming Unit – Macrophage).

Mass spectrometry

Proteomic analyses were performed at the Institute for Research in Immunology and Cancer (IRIC), University of Montréal. Tryptic digest of the EV samples (n=6 males and n=6 females) were analyzed by LC-MS/MS analyses as follows. Briefly samples were loaded on an Optiguard C18 precolumn and separated on a home-made reversed-phase column (150-µm i.d. by 150 mm). Each full MS spectrum acquired at a resolution of 60,000 was followed by 15 tandem-MS (MS-MS) spectra on the most abundant multiply charged precursor ions. Tandem-MS experiments were performed using collision-induced dissociation (HCD) at a collision energy of 27%. The data were processed using PEAKS X (Bioinformatics Solutions, Waterloo, ON) and a human database. Mass tolerances on precursor and fragment ions were 10 ppm and 0.01 Da, respectively. Intensities were normalized based on the total ion current and the median expression calculated and combined per age group. Intensities were normalized based on the total ion current and the median expression calculated and combined per age group.

Bioinformatics

Data preprocessing, filtering, and subsequent analyses were performed in MATLAB (Mathworks, Inc., MA, USA, version R2019a). To identify sample outliers and/or sequencing batch effects, the expression profiles were assessed through visual inspection and correlation analysis as described (Panarelli, N. et al. (2019) Endocr. Relat. Cancer 26, 47–57). To assess protein abundance between young, middle and older groups, the proteins were ranked based on the median level of expression with each group. To identify

proteins that discriminate between three age groups, we used an established feature selection algorithm (Ren, R. et. al (2017). Oncotarget 8, 70982–71001) with leave-one-out cross-validation. From the top 5% of ranked proteins from each age group against all other samples 31 proteins were selected as the best discriminators and were visualized by hierarchical clustering using complete linkage and Euclidean distance for rows (proteins) and Spearman correlation for columns. The selected proteins were also used to show the age group clustering using t-Distributed Stochastic Neighbor Embedding (t-SNE)and Principal component analysis (PCA) plots. Kruskal Wallis -test was used to assess gender differences for the selected proteins. The top 5% of ranked proteins were used to identify the tissue of origin by linking with the human protein atlas (Uhlen, M. et al. (2015) Science 80- 347, 1260419–1260419) for each age group.

Flow Cytometry Analysis of $CD34^{+}CD38^{-}$ cells

Post 48-hour incubation with EVs, CD34+ cells were washed (PBS/2% fetal bovine serum (FBS)) by centrifuging at 300g / 10 min / RT. Cells were incubated with fluorescently conjugated antibodies for 45 min on ice followed by washing cells in cold PBS/2% FBS and centrifuging at 300g / 10 min /4°C. Antibodies include: mouse-anti-human CD34 (Fluorescein Isothiocyanate (FITC), clone 581, BD Biosciences), mouse-anti-human FITC isotype (FITC, clone RMG1-1 Biolegend), mouse-anti-human CD38 (Allophycocyanin (APC), clone HIT2, BD Biosciences), mouse-anti-human APC Isotype (APC, clone P3.6.2.8.1, eBiosciences). Cells were analyzed using a CytoFLEX-S flow cytometer (Beckman Coulter, USA). Analysis of flow cytometry data was performed using FlowJo Software.

Electron microscopy

For the negative-staining preparation, approximately 5µL of concentrated EV sample were loaded onto mesh carbon film grids (with glow discharge). The sample was left to adsorb onto the grid for 5 minutes, rinsed with distilled water, and stained with 2% uranyl acetate/H2O for 1min. After the sample preparations were air dried, they were examined using a JEM-2100 Electron Microscope with the follow settings: 80KV, Magnification is 50K to 80K.

Bead-based flow cytometry analysis of extracellular vesicle markers

To select for tetraspanin positive populations, anti-CD9, anti-CD63, or anti-CD81-coated superparamagnetic polystyrene beads (4.5µm) (Dynabeads®, Thermo Fisher Scientific) were used as per manufactures instructions (Thermo Fisher Scientific MAN0007670). For counterstaining of the captured extracellular vesicle populations, phycoerythrin (PE)-conjugated anti-CD9 antibody (mouse anti-human

clone H19a) was used and compared with isotype-matched negative control (PE-mouse anti-human clone MOPC-21). Cells were analyzed using a CytoFLEX-S flow cytometer. Analysis of flow cytometry data was performed using FlowJo Software.

Western blot

To demonstrate the presence of protein markers associated with EVs, equal EV protein from young, middle-aged and old subjects were loaded and ran on 10% Bis-Tris polyacrylamide gels. For detection of CD63, non-reducing conditions were used. Membranes were blocked for 1 hour using 5% non-fat dry milk in 1X TBS-T (Tris Buffered Saline with Tween 20). Membranes were probed with anti-CD63 antibody (Purified mouse anti-human CD63; BD PharmingenTM) overnight at 4°C. Blots were developed using ECL luminol-based enhanced chemiluminescent substrate (UltraScence Pico Ultra Western Substrate; FroggaBio) and visualized using Azure c600 imaging system.

Apoptosis assay

After incubation with or without extracellular vesicles, CD34$^+$ cells were stained with annexin-V (Allophycocyanin APC (Biolegend)) and DAPI (Sigma-Aldrich) in the presence of Hank's balanced salt solution (HBSS) (Millipore-Sigma) for 20 min at room temperature. Cells were immediately analyzed using a CytoFLEX-S flow cytometer. Analysis of flow cytometry data was performed using FlowJo Software Results are expressed as a mean ± SD.

Statistical analysis

Data analysis and graphical presentations were performed using GraphPad Prism version 8.0 (GraphPad Software, USA). Statistical analysis for the characterization of blood extracellular vesicles in terms of median size, protein concentration, and particle concentration between sex (male n=18, female n=17) was conducted using an unpaired t-test, and between age groups (young n=12, middle-aged n=11, older n=12) using ordinary One-Way ANOVA. CFC statistical analysis was conducted using mixed-effects analysis, with the Geisser-Greenhouse correction, along with Tukey's multiple comparisons test with individual variances computed for each comparison. Statistical analysis of blood extracellular vesicles in terms of median size, protein concentration, and particle concentration plotted against donor age included linear regression analysis (n=35). Data is displayed as mean ± SEM unless stated otherwise. Significant statistical differences (* $p<0.05$, ** $p<0.01$, ***$p<0.001$) are indicated unless otherwise noted.
